# Feasibility of conducting brand-specific influenza vaccine effectiveness studies in three Nordic countries, Denmark, Finland, Sweden

**DOI:** 10.2807/1560-7917.ES.2026.31.8.2500648

**Published:** 2026-02-26

**Authors:** Kristyna Faksova, Emilia Myrup Thiesson, Nicklas Pihlström, Ulrike Baum, Eero Poukka, Tuija Leino, Rickard Ljung, Anders Hviid

**Affiliations:** 1 Department of Data Science and AI in Health, Statens Serum Institut, Copenhagen, Denmark; 2 ECDC Fellowship Programme, Field Epidemiology path (EPIET), European Centre for Disease Prevention and Control (ECDC), Stockholm, Sweden; 3 Division of Use and Information, Swedish Medical Products Agency, Uppsala, Sweden; 4 Department of Public Health, Finnish Institute for Health and Welfare, Helsinki, Finland; 5 Department of Public Health, Faculty of Medicine, University of Helsinki, Helsinki, Finland; 6 Institute of Environmental Medicine, Karolinska Institutet, Stockholm, Sweden

**Keywords:** seasonal influenza vaccine, vaccine effectiveness, Nordic countries, registries, target trial emulation framework, cohort studies, test-negative case-control design, robust post-authorisation

## Abstract

Annual reformulation and approval of seasonal influenza vaccines necessitate yearly evaluation of their effectiveness. Regulatory agencies, including the European Medicines Agency (EMA), rely on timely, real-world evidence to inform product-specific benefit-risk assessments. We explored the feasibility of conducting annual, brand-specific influenza vaccine effectiveness studies in Denmark, Finland and Sweden, starting with the 2024/25 season. These countries maintain population-wide vaccination, clinical and laboratory registers, linkable via personal identification numbers and updated in near real-time. We discuss suitable study designs and document that cohort studies using a target trial emulation (TTE) framework are feasible in all three countries; register-based test-negative case-control design (TND) studies are currently only feasible in Denmark. Supplementary methods, including regression discontinuity and negative control outcome analyses, can address residual bias. This Nordic collaboration has proven capacity for large-scale register-based studies and its infrastructure is able to address EMA’s requirements for timely, robust post-authorisation evidence to guide public health and regulatory decisions.

## Background

Seasonal influenza vaccines are reformulated annually to match circulating influenza strains following recommendations issued during the biannual World Health Organization (WHO) influenza vaccine strain selection meetings. As vaccine composition may vary each season, vaccine effectiveness must therefore be established annually. The immunogenicity trials that support licensure are typically small and cannot reliably predict how individual vaccine brands will perform across diverse age and risk groups. This leaves vaccine regulators without timely evidence for product-specific benefit-risk assessments. The European Medicines Agency (EMA) guidelines on influenza vaccines state that the optimal way to monitor the annual performance of influenza vaccines is to generate high-quality evidence through non-interventional, brand-specific influenza vaccine effectiveness (IVE) studies in real-world settings [1]. In addition to generating annual evidence on effectiveness, findings from IVE studies may also highlight issues related to the quality of an individual vaccine, that may be due, for example, to manufacturing changes, and require prompt, targeted regulatory action. From a public health perspective, National Immunization Technical Advisory Groups (NITAGs) and public health authorities are primarily interested in the overall performance of vaccination strategies based on the vaccine products available at country or regional level. While robust brand-specific evidence may contribute to national decision-making and public health recommendations, vaccine selection and procurement are primarily informed by vaccine type and programmatic considerations. The focus of this manuscript is to inform the feasibility of IVE studies to support the regulatory strategy in future influenza seasons under the European Vaccine Monitoring Platform [2].

The availability of national vaccination, laboratory and clinical registries in the three Nordic countries of Denmark, Finland and Sweden, linkable through personal identification numbers, updated in near real-time and covering more than 22 million residents, have the capacity to facilitate such IVE studies [2]. This perspective assesses the feasibility of the Nordic register and analytical infrastructure to support annual, brand-specific IVE studies beginning with the 2024/25 influenza season. It focuses on vaccines used in older adults aged 65 years and older, among whom enhanced formulations such as adjuvanted and high dose products have recently been introduced in several Nordic countries and warrant evaluation of real-world effectiveness. Moreover, it synthesises the detailed, technical feasibility assessment registered in the Heads of Medicines Agencies (HMA)–EMA Catalogue for surveillance and public health teams [3].

### Vaccine effectiveness monitoring and evaluation in the Nordic countries

During the COVID-19 pandemic, the larger Nordic countries - Denmark, Finland, Norway and Sweden - collaborated on large-scale studies evaluating the effectiveness and safety of COVID-19 vaccines [4-7]. These efforts produced rapid, high-impact evidence that informed public health and regulatory decision-making at national, regional and international level. Nordic studies were among the first to assess key vaccine safety signals, including the association between viral vector vaccines and thromboembolism with thrombocytopenia syndrome [8] and between mRNA vaccines and myocarditis [6], directly influencing national vaccination policies [7].

In the context of regulatory decision-making at EMA, the three Nordic countries Denmark, Finland and Sweden jointly conducted comprehensive influenza vaccine effectiveness (VE) evaluations during the 2024/25 influenza season [9]. The study, commissioned by EMA following a competitive tender process, and conducted in close collaboration with EMA, demonstrated the feasibility of rapid effectiveness research in the Nordic setting for both newly introduced and seasonal vaccines. Three Nordic countries (Denmark, Finland and Sweden) share a common welfare model that provides universal, tax-funded healthcare to their populations.

### Settings and data sources for conducting brand-specific influenza vaccine effectiveness studies

**Figure 1:**
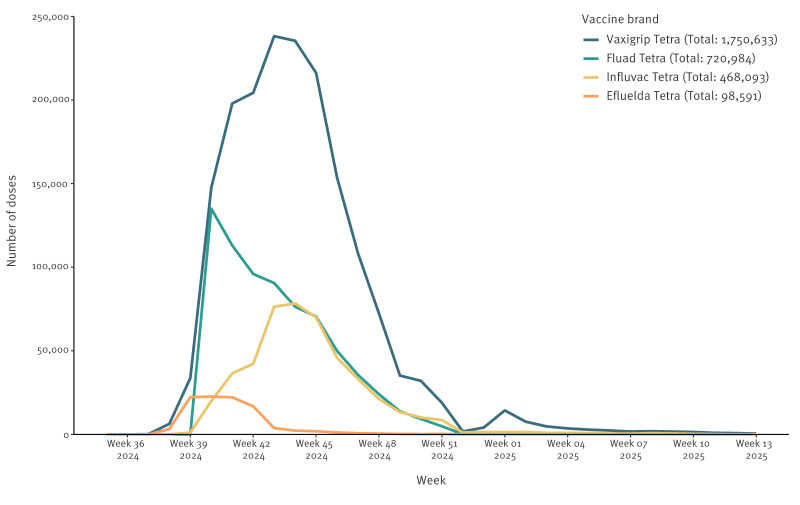
Weekly dose uptake of the four most frequently administered influenza vaccine brands in Denmark, Finland and Sweden (2 regions), 2024/25 influenza season

Across Denmark, Finland, and Sweden, IVE studies are ethically feasible as they are conducted within established national surveillance frameworks or approved register-based research, which do not require individual informed consent, with country-specific legal and ethical conditions detailed in Supplementary Table S1. Each country operates a wide range of government-maintained population-based registries containing individual-level data on demographics and healthcare [10]. Each resident is registered with a unique personal identifier, implemented as part of the national civil registration systems established between 1964 and 1969 [3,4]. This identifier enables individual-level data linkage across various registries allowing researchers to pool data into a large study cohort, which enhances statistical power and enables robust research with less selection bias over longer periods [7]. A key feasibility advantage of this infrastructure is timeliness. Vaccination and infectious disease surveillance registers are updated regularly, enabling the generation of interim IVE estimates within 5–6 weeks of season onset. For the purpose of estimating IVE, only PCR-confirmed influenza cases are used to ensure consistent and high sensitivity case ascertainment across countries. These near real-time analyses can provide brand-specific IVE estimates to inform early regulatory decision-making. Figure 1 presents cumulative influenza vaccine brand uptake across Denmark, Finland and Sweden during the 2024/25 influenza season.

Denmark operates a robust national influenza surveillance system overseen by the Statens Serum Institute (SSI) [11]. The Statens Serum Institute integrates nationwide influenza surveillance with the Danish vaccination register, which has captured every influenza vaccination dose since reporting became mandatory on 15 November 2015. Each entry records vaccination date and commercial brand, enabling complete, brand-specific exposure ascertainment [12].

To carry out national influenza surveillance in Finland, the Finnish Institute for Health and Welfare combines traditional virological surveillance with the analysis of electronic health records, including a vaccination register. Primarily derived from primary care visit data, the vaccination register stores vaccination date, commercial brand and batch number for almost all doses; only data on vaccinations in some long-term care settings may be missing [13,14].

The Swedish Public Health Agency oversees national influenza surveillance; brand-level vaccination data are currently available from two regions - Uppsala and Blekinge - covering ca 563,000 residents. Including seasonal influenza vaccination in the government-mandated national vaccination register is currently under discussion [15].

A comprehensive overview of the available country-specific data sources is provided in Supplementary Table S2. Tables 1 and 2 below describe the characteristics of vaccination and hospital registry data as well as the features of infectious disease surveillance in Denmark, Finland and Sweden. Table 3 illustrates how these characteristics address known methodological challenges of IVE studies.

**Table 1:** Characteristics of vaccination registry data for influenza vaccine effectiveness studies, Denmark, Finland and Sweden [3]

**Completeness**	The vaccination registers in Denmark, Finland, and Sweden provide varying levels of completeness. - Denmark's register contains nationwide data on all vaccinations since 2015, including privately funded vaccinations if reported. - Finland’s register covers vaccinations from the public primary care sector, private and occupational sectors (since 2021), and secondary care (since 2019), though social caregiver-administered vaccinations (e.g. in nursing homes) may be incompletely covered. - Sweden’s national vaccination register was implemented in 2013 but only mandates reporting for vaccinations included in national immunisation programmes (e.g. childhood vaccinations, COVID-19 vaccines). Seasonal influenza vaccinations are not yet comprehensively recorded at the national level but are captured regionally (currently two regions included).
**Selection bias**	The universal healthcare systems in Denmark, Finland and Sweden ensure equal access to publicly funded vaccinations, reducing the risk of selection bias related to socioeconomic factors such as income level or employment status.
**Information bias**	In all three countries, vaccination registers provide high-quality data, but potential misclassification of vaccination status may occur due to reporting delays or errors by healthcare providers. - In Denmark and Finland, minor inconsistencies in recording batch numbers and vaccine brand names have been noted for earlier years, though these issues have improved over time. - In Sweden, data on influenza vaccinations is only available regionally, but this is unlikely to introduce information bias.
**Confounding**	The Nordic vaccination registers can be linked to other national registries (e.g. hospital, prescription and demographic registers) using unique personal identifiers, enabling control for confounders such as comorbidities, healthcare-seeking behaviour and vaccination history. However, unmeasured confounders, such as lifestyle factors may still pose challenges in non-interventional studies.

**Table 2:** Characteristics of hospital and infectious disease surveillance data for influenza vaccine effectiveness studies, Denmark, Finland and Sweden [3]

**Completeness**	The Nordic hospital care registers capture comprehensive data on in-hospital care, with coverage spanning several decades. These registers provide near-complete health histories for specialised care, supporting life-course epidemiology across large populations. Private hospital data may be less consistently included, and milder primary care influenza cases are not captured in hospital-based datasets. Infectious disease surveillance systems in the region also provide national coverage of laboratory-confirmed influenza cases, though variations in testing practices can affect completeness.
**Selection bias**	The population-based nature of the Nordic healthcare systems, with universal access, minimises selection bias related to socioeconomic factors, insurance status, or care-seeking behaviours. However, surveillance systems relying on laboratory-confirmed cases may introduce bias related to testing behaviours and recommendations.
**Information bias**	Misclassification of outcomes may arise from inaccuracies in diagnostic coding or delays in receiving laboratory results. This is particularly relevant for older patients, where the initial diagnosis may not include influenza if symptoms are atypical or masked by chronic conditions. While Nordic registers are generally reliable, there may be under-reporting of private healthcare services or self-funded treatments. Testing strategies for influenza also differ across regions and seasons, which leads to incomplete reporting of influenza cases, especially during periods of low testing.
**Confounding**	Nordic health data systems enable linkage of hospital care registers with vaccination, population, and prescription registers using unique personal identifiers. This facilitates control for many confounders, such as comorbidities, healthcare-seeking behaviour, and vaccination history. However, unmeasured confounders, such as frailty, and general health status may still pose challenges.

**Table 3:** Overview of strengths and limitations of suitable study designs and supplementary analyses for estimating seasonal influenza vaccine effectiveness [3]

**Study design**	**Strengths**	**Limitations**	**Feasibility**
**Cohort design using the target trial emulation (TTE) framework**	Mimics randomised controlled trial design using observational data; can reduce bias if appropriately designed; provides an interpretable causal framework.	Susceptible to unmeasured confounding; potential biases from healthcare access and healthcare-seeking behaviours; methodologically complex.	Feasible with granular data; relies on high data quality and completeness.
**Test-negative design (TND)**	Aims to reduce bias from healthcare-seeking behaviour; timely VE estimation during influenza season; well-established method for respiratory VE studies.	Assumes influenza vaccination does not modify the effectiveness of vaccines targeting other respiratory pathogens; potential collider bias; cannot be used for all-cause outcomes.	In the Nordic context, the TND is implemented using routinely collected registry data rather than active clinical enrolment. Consequently, clinical symptom information and precise timing of specimen collection relative to illness onset are not available, which differentiates this register-based TND from traditional sentinel surveillance designs. This design is feasible in Denmark, not feasible in Finland and Sweden due to unavailability of negative influenza test results.
**Supplementary analyses**	**Strengths**	**Limitations**	**Feasibility**
**Prior event rate adjustment**	Adjusts for pre-vaccination differences in outcome rates, providing a valid causal estimate under the assumption of stable relative differences in outcome rates before and after vaccination among the comparison groups.	Limited power due to the rarity of influenza outcomes outside the season; potential confounding from cross-reactive immunity and infection-induced immunity; susceptible to calendar time-related changes in healthcare seeking behaviour.	Feasible, but limited power due to seasonal variability of influenza outcomes.
**Regression discontinuity analysis**	Provides valid causal estimate when vaccination eligibility is based on age; suitable for simple intention-to-treat analysis of individuals just above and below age threshold.	Limited generalisability to broader population; requires strict adherence to age thresholds; potential issues with statistical power.	Feasible with access to age-specific vaccination data in registers.
**Negative control outcomes analysis**	May detect residual confounding by examining outcomes (e.g. lower back pain, clavicle fracture, diverticulitis) presumably unrelated to vaccination. Null findings support validity of VE estimates.	Potential violations of the assumptions of the outcome being causally unrelated to vaccination and that any association with vaccination reflects the same confounding mechanism present in the association between vaccination and influenza. Rare outcomes may be less suitable due to limited precision.	Feasible if these diagnoses are well-coded in national registers.

TND: test-negative design; TTE: the target trial emulation framework; VE: vaccine effectiveness.

## Study designs for estimating brand-specific influenza vaccine effectiveness

The current state-of-the-art in non-interventional studies estimating VE are (i) the cohort study design using the target trial emulation (TTE) framework and (ii) the test-negative case-control design (TND). Both approaches seek to mitigate the impacts of bias and confounding [16].

The TTE framework is recommended by EMA as a strategy to formalise the design and analysis of non-interventional studies with causal objectives [17]. This recommendation reflects both methodological rigour and the current data landscape, in line with EMA guidance that recognises TTE as a structured framework for causal inference in non-interventional studies [17]. Under the TTE framework, investigators specify the protocol elements of a hypothetical randomised controlled trial - eligibility, treatment strategies, follow-up, endpoints and analytic contrasts - and then emulate that trial using real-world data. This approach provides transparency, minimises immortal-time bias and yields interpretable measures of effectiveness [18].

The TND restricts the study population to people who seek influenza testing when presenting with influenza-like illness and compares vaccination odds from test-positive cases and test-negative controls. By placing conditions on testing, TND aims to mitigate healthcare-seeking bias, but may also introduce collider bias in the association between vaccination and outcome [19]. The test-negative design cannot yet be implemented consistently across all Nordic countries due to incomplete availability of influenza test-negative results.

Guilin Li and colleagues [16] compared the performance of the two study designs for estimating COVID-19 VE using registry data. Where extensive covariate data were available, both approaches yielded similar VE estimates. However, with more limited covariate information, reducing the ability to account for healthcare-seeking behaviour, TND tended to produce higher VE estimates and TTE tended to produce lower ones [16]. The methodological aspects and feasibility of these study designs are further described in Table 3.

### Illustrative findings from the 2024/25 influenza season

To complement the methodological considerations described above, and in response to the data availability for the 2024/25 influenza season, we present a concise summary of illustrative IVE estimates obtained using the Nordic registry infrastructure published in [9]. These examples demonstrate how the Nordic infrastructure performs in practice and highlights the operational and analytical constraints encountered during the first season of implementation. The purpose of presenting these findings is not to provide a full evaluation of VE, but to illustrate feasibility, data availability and the practical implications of differential vaccine brand uptake, national vaccination policies and the varying completeness of test and follow-up data across the Nordic countries.

**Figure 2:**
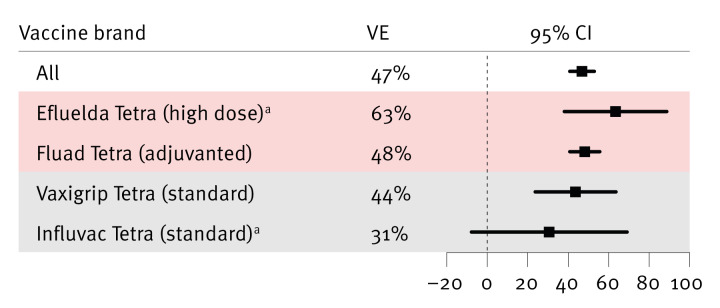
Influenza vaccine effectiveness estimates against hospitalisation: comparison of seasonal vaccine recipients with non-recipients, Denmark and Finland, the 2024/25 influenza season

CI: confidence interval; VE: vaccine effectiveness.

^a^ Only estimated in Denmark.

Brand-specific IVE analysis was conducted in Denmark and Finland during the 2024/25 season (Figure 2). Influenza VE estimates were reported in detail in our analytical study [9] and are summarised here briefly to illustrate feasibility and the performance of the Nordic infrastructure. Sweden was unable to contribute comparable estimates because follow-up data were not available for the full duration of the season. Vaccine effectiveness was calculated in each country as 1 − the risk ratio for influenza-related hospitalisation among vaccinated versus unvaccinated individuals, and country-specific estimates were subsequently pooled using a random-effects meta-analysis with restricted maximum likelihood estimation [9]. Among adults aged 65 years or older, the estimated IVE against influenza-related hospitalisation during week 18 2025 (based on data availability) was 47% (95% confidence interval (CI): 41% to 53%) overall. Vaccine brand-specific estimates were 63% (95% CI: 38% to 88%) for Efluelda Tetra (high dose) and 31% (95% CI: −8% to 69%) for Influvac Tetra (standard dose) - both administered in Denmark only - and 48% (95% CI: 41% to 56%) for Fluad Tetra (adjuvanted) and 44% (95% CI: 24% to 66%) for Vaxigrip Tetra (standard dose). These IVE estimates were obtained using the TTE framework. In Denmark, where test-negative data were available, the TTE and TND estimates for laboratory-confirmed influenza infection were highly consistent (39.7% vs 39.5%, respectively), demonstrating alignment between the two analytical approaches.

VE estimation on day 126, week 18 2025.

These initial findings illustrate both the feasibility and the inherent limitations of producing brand-specific IVE estimates in the Nordic setting. Several factors constrained the precision and comparability of these estimates: (i) age- and indication-based vaccine allocation, (ii) country-specific vaccine availability, (iii) limited sample sizes and statistical precision, (iv) challenges of stratified analysis, and (v) data availability constraints.

#### Age- and indication-based vaccine allocation

Vaccine brand allocation was widely age dependent. In Denmark, adjuvanted and high dose vaccines were offered to individuals aged 70 years or older, whereas in Finland these products were targeted to those aged 85 years or older. In contrast, younger age groups primarily received non-adjuvanted standard dose vaccines. An overview of the vaccine brands and their target populations in each of the three Nordic countries is presented in Supplementary Table S3. These systematic differences in target groups introduce selection bias, as apparent differences in IVE between brands may reflect differences in age, frailty and underlying health status rather than vaccine performance. Moreover, these allocation patterns also influence the expected direction of bias: enhanced, such as adjuvanted and high dose, vaccines are primarily administered to elderly persons (e.g. 70 years and older) who are more affected by immunosenescence, meaning that their observed VE is likely an underestimate of what would be seen in younger individuals aged 65–69 years. Conversely, standard dose vaccines are primarily given to those 65-69-years-old. If administered to older and frailer individuals, their VE would likely be lower. As a result, any theoretical head-to-head comparison would likely show a larger difference between vaccine types than the indirect, target group-specific comparisons possible under current allocation structures.

#### Country-specific vaccine availability

Certain brands, for example, Efluelda Tetra (high dose) and Influvac Tetra (standard dose), were used only in Denmark, limiting comparable IVE estimates across countries. More harmonised vaccination recommendations and brand use across the Nordic countries would facilitate comparison and allow more robust cross-country assessments of brand-specific IVE.

#### Limited sample sizes and statistical precision

The statistical precision of brand-specific IVE estimates depended on the number of doses administered. Brands with lower uptake produced wider CIs, particularly when used in a single country (Figure 2). Although comparative analysis between brands could, in principle, be undertaken by matching recipients by age and other key characteristics, the limited overlap across these characteristics and low coverage of certain vaccine brands constrains this approach. As a result, head-to-head comparisons would be underpowered.

#### Challenges of stratified analysis

The diversity and uneven use of influenza vaccine products across countries limits the ability to conduct further stratified analysis, for example, by influenza subtype, age group, underlying conditions or time since vaccination. While such stratification is relevant for public health decision-making, it increases the risk of sparse data bias and unstable estimates.

#### Data availability constraints

Sweden was unable to contribute IVE estimates for the same follow-up period as Denmark and Finland. In addition, Finland and Sweden lack test-negative results, limiting the consistent application of the TND.

### Supplementary analysis to minimise biases

To evaluate the potential for bias by healthcare-seeking behaviour or healthcare access in TND and TTE studies, several supplementary analytical designs can be applied. These include prior event rate ratio (PERR) adjustment, regression discontinuity analysis (RDA) and negative control outcomes analyses. Such approaches enable contextualisation of results and may improve the robustness of findings. Moreover, triangulation - integrating evidence from multiple analyses with different methodologies - can further enhance the interpretation of results and identify residual biases. In Table 3 we summarise the strengths, limitations and feasibility of these study designs in Denmark, Finland and Sweden.

### Regulatory and public health policy context

The results of this Nordic collaboration have the capacity to address the requirements of the EMA guidance on influenza vaccines by delivering population-level evidence that is comprehensive, timely and reproducible. Brand-specific IVE estimates allow regulators to assess the annual performance of seasonal influenza vaccines and inform their benefit-risk profile. From a public-health perspective, brand-level VE estimates provide complementary insight into how well individual vaccines protect the specific populations for which they are recommended. Such information can help determine whether available products offer sufficiently effective protection for older adults and other risk groups. Furthermore, they can inform equitable distribution of the most effective types of products across high-risk groups, particularly where vaccine supply or procurement contracts limit access. In settings with brand heterogeneity or constrained resources, these estimates can support evidence-based prioritisation. While the present feasibility assessment focused on seasonal influenza vaccination, the register-based infrastructure of the Nordic countries is flexible and could be rapidly adapted to monitor effectiveness of pandemic or outbreak-response vaccines (e.g. avian influenza or future severe acute respiratory syndrome coronavirus 2 (SARS-CoV-2) variants). Building and maintaining this Nordic analytical infrastructure strengthens overall European Union (EU) vaccine preparedness capacity. In addition, the findings of this feasibility assessment may inform the European Vaccine Monitoring Platform’s endeavours to expand feasibility beyond the Nordic countries and to other types of data collection and data sources. This could help develop the capacity to assess the effectiveness of vaccines brands across the EU/European Economic Area (EEA), as not all available brands are used in the Nordic countries [2].

## Conclusions

This feasibility assessment shows that the Nordic health data infrastructure can support timely and robust brand-specific IVE evaluations on an annual basis. The assessment confirms that the Nordic registry and analytical infrastructure provide a structured approach for generating interpretable, causal estimates from routinely collected data.

At the same time, several challenges remain. Precision and comparability are influenced by heterogeneous vaccine uptake, country-specific recommendations, age-dependent allocation patterns and small sample sizes for some products. In addition, the lack of test-negative data in Sweden and Finland and the limited national coverage of vaccination data in Sweden constrain the consistent use of TND and a full nationwide IVE analysis. These factors highlight the need for harmonised definitions, coordinated analytic timelines, and, in some situations, pooled analyses to strengthen statistical power and comparability across countries.

Target trial emulation analysis remains feasible across Denmark, Finland and Sweden, and supplementary approaches such as PERR adjustment, regression discontinuity and negative control outcome analyses can help address potential biases, including healthcare-seeking behaviour and residual confounding. The proven ability of the Nordic countries to conduct high-quality collaborative VE studies demonstrates their readiness to support a sustainable programme of annual brand-specific IVE evaluations. The Nordic infrastructure can meaningfully inform public health and regulatory decision-making and contribute to broader EU/EEA vaccine preparedness.

## Data Availability

The individual-level dataset analysed in this study cannot be shared publicly because legal, ethical, and IT-security restrictions prohibit transfer outside the custodial institution. Since the work is carried out under national surveillance activities in the respective countries, using patient identification information without individual patient consent as part of the legal requirement for public health surveillance and monitoring, authors cannot make the underlying dataset publicly available for ethical and legal reasons.

## Supplementary Data

244000 Supplementary Material
