# Supplementary material for: Feasibility of conducting brand-specific influenza vaccine effectiveness studies in three Nordic countries, Denmark, Finland, Sweden
Source: Euro Surveill. 2026 Feb 26;31(8):2500648. doi: 10.2807/1560-7917.ES.2026.31.8.2500648 (PMC13074543; doi:10.2807/1560-7917.ES.2026.31.8.2500648)
Supplement: Supplementary Material [file 25-00648_FAKSOVA_Supplement.pdf]

Supplementary Appendix

This supplementary material is hosted by *Eurosurveillance* as supporting information alongside the article 'Feasibility of conducting brand-specific influenza vaccine effectiveness studies in three Nordic countries, Denmark, Finland, Sweden', on behalf of the authors, who remain responsible for the accuracy and appropriateness of the content. The same standards for ethics, copyright, attributions and permissions as for the article apply. Supplements are not edited by *Eurosurveillance* and the journal is not responsible for the maintenance of any links or email addresses provided therein.

Table of contents

Supplementary Table S1. Ethics approval/exempt within each country. 1

Supplementary Table S2. Overview of available national registries. 2

Supplementary Table S3. Overview of vaccine brands used in the national program in the Nordic countries in season 2024/2025. 5

Supplementary References 5

Supplementary Table S1. Ethics approval/exempt within each country.

Country	Ethical Regulations
Denmark	The Danish analyses are performed as surveillance activities analyses as part of the advisory tasks of the governmental institution Statens Serum Institut (SSI) for the Danish Ministry of Health. SSI's purpose is to monitor and fight the spread of disease in accordance with section 222 of the Danish Health Act. According to Danish law, national surveillance activities conducted by SSI do not require approval from an ethics committee. Both the Danish Governmental law firm and the compliance department of SSI have approved that the study is fully compliant with all legal, ethical, and IT-security requirements and there are no further approval procedures required for such studies.
Finland	By Finnish law, the Finnish Institute for Health and Welfare (THL) is the national expert institution to carry out surveillance of the impact of vaccinations in Finland (Communicable Diseases Act, https://www.finlex.fi/en/laki/kaannokset/2016/en20161227.pdf). Neither specific ethical approval (a waiver of ethical approval was received from Chief Doctor Otto Helve, Director of the Department for Health Security Finnish Institute for Health and Welfare) of this study nor informed consent from the participants are needed.
Sweden	The Swedish analyses are conducted under the Swedish Ethical Review Authority approval 2020-06859, 2021-02186 and conformed to the principles embodied in the Declaration of Helsinki. Register-based studies (like this) in Sweden are exempt from obtaining consent to participate.

Supplementary Table S2. Overview of available national registries.

Country	Data sources						
**Denmark**							
**Title**	**Info**	**Type**	**Setting**	**Study availability**	**Update**	**Lag**	**Ref**
The Danish Civil Registration System	The register provides the unique personal identifier for all permanent residents of Denmark that allows linkage between all Danish health care registers and civil registrations systems. In addition, it holds general demographic information such as birthdate and sex as well as continuously updated information and dates on historical addresses, immigration and emigration status, and death.	Register	Nationwide	1968- today	Daily	No lag	(1)
The Danish vaccination register	The register holds information on all vaccinations given in Denmark including information on vaccination date, brand, type, dose, and product batch number ever since November 15, 2015 (when reporting to the register became mandatory).	Register	Nationwide	2020 – today	Daily	No lag	(2)
The National patient registry	The register covers all hospital contacts/visits in Denmark with information on the duration of the contact/visit, department of admission and other hospital characteristics. Treating physician-assigned diagnoses have been registered according to ICD-10 codes since 1995.	Register	Nationwide	1995 - today	Daily	No lag	(3)
The Danish Microbiology Database	Information on positive results of RT-PCR tests for influenza are obtained from The Danish Microbiology Database (MiBa) which holds information on all microbiology samples analysed at Danish departments of microbiology, including information on influenza test results, date of sampling, date of analysis, type of test and interpretation of the test (positive / negative).	Register	Nationwide	2020 – today	Daily	No lag	(4)

Country	Details of the individual-level data sources						
**Finland**							
**Title**	**Info**	**Type**	**Setting**	**Study availability**	**Update**	**Lag**	**Ref**
Finnish Population Information System	The register is held by the Digital and Population Data Services Agency and contains personal data on all permanent residents in Finland such as the unique personal identifier, date of birth, place of residence, date of death, and date of immigration, and emigration.	Register	Nationwide	1964 - today	Daily	No lag	(5)

National Vaccination Register	The register, which is based on the Register of Primary Health Care Visits, holds information on almost all influenza vaccinations administered in Finland; only influenza vaccinations given by social care givers such as nursing homes might be incompletely covered. Data include the date of vaccination, vaccine batch number and trade name.	Register	Nationwide	2009 - today	Daily	No lag	(6)
Care Register for Health Care	The register comprises information on all in-hospital care (since 1969) and outpatient specialist care (since 1998) in Finland, including admission and discharge dates, whether hospitalisation was planned or acute, codes for discharge diagnoses (according to ICD-10) and surgical procedures, whether discharged as deceased, to own private residence or other health care facilities, type of department and hospital. The register is held by the Finnish Institute for Health and Welfare.	Register	Nationwide	1967 - today	Daily	1-4 weeks	(7)
Register for Primary Health Care Visits	The register is held by Finnish Institute for Health and Welfare and holds data on all primary health care services delivered in Finland.	Register	Nationwide	2011 – today	Daily	No lag	(8)
National Infectious Diseases Register	The register contains information on notifiable diseases which must be reported by the laboratories and the physician treating the patient, or performing an autopsy, in accordance with the Finnish Communicable Diseases Act. All laboratory-confirmed influenza infections are recorded in the National Infectious Diseases Register. The register is held by the Finnish Institute for Health and Welfare.	Register	Nationwide	1995 - today	Daily	0-1 weeks	(9)
Special Reimburseme nt Register and Prescription Centre database	The Special Reimbursement Register holds information on individuals entitled to special reimbursement for medical expenses. The Prescription Centre database holds information on individuals using selected medications of interest. These databases are maintained by the Finnish Social Insurance Institution.	Register	Nationwide	1995 – 2023	Every 6 months	0–6 months	(10)
Finnish Intensive Care Quality Register	The register includes all intensive care admissions with primary diagnosis (ICD-10).	Register	Nationwide	2020 – today	Daily	No lag	(11)

**Country**	**Details of the individual-level data sources**						
**Sweden**							
**Title**	**Info**	**Type**	**Setting**	**Study availabilit y**	**Updat e**	**Lag**	**Ref**

Swedish vaccination register	The register will contain information on administered influenza vaccines including data on the date of administration, the specific vaccine products, substance, formulation, batch number and dose number (for repeated doses). The register is held by the Public Health Agency of Sweden.	Register	Nationwide	2026-onwards	Daily	No lag	(12)
Regional vaccination data	Regional data contains information on administered influenza vaccines including data on the date of administration, and the specific vaccine products.	Regional data	Regional	2020-	Ad hoc		
Swedish national inpatient register	The register comprises information on all in-hospital (since 1987) and out-patient (since 2001) specialist care in Sweden including data on admission and discharge dates, whether hospitalisation was planned or acute, codes for discharge diagnoses and surgical procedures, whether discharged as deceased, to own private residence or other health care facilities, type of department, and hospital. For the current study period discharge diagnoses were recorded according to the Swedish clinical modification of the ICD-10 (i.e. ICD-10-SE). The register is held by the National Board of Health and Welfare.	Register	Nationwide	2017 - today	Monthly	2–4 week	(13)
Swedish Prescribed drug register	The Swedish Prescribed Drug Register contains details of all the prescriptions dispensed in Sweden since July 1, 2005. It is updated monthly with around 100 million prescriptions dispensed each year. It covers the entire Swedish population and includes information on unique personal identifier of the patient, age, sex, place of residence, and prescription information on substance, brand name, formulation and package dispensed amount, dosage (in free text) and unique expenditure and reimbursement, date of prescribing and dispensing, practice that has issued the prescription, and prescriber's profession. Drugs are identified by a unique identifier for each specific combination of brand name, substance, formulation, and package. Additionally, all drugs are classified according to the Anatomic Therapeutic Chemical Classification System (ATC). The register only includes filled prescriptions, not medicines sold over the counter, nor medicines administered directly by health-care personnel without prescription. The register is held by the National Board of Health and Welfare.	Register	Nationwide	2017	monthly	2 weeks	(14)
Register on Surveillance of Notifiable Communicable Diseases (Sminet)	The register contains information on notifiable diseases (for which reporting is mandatory) reported by either the analysis-performing laboratories, the treating physician or the autopsy-performing physician, in accordance with the Swedish Communicable Diseases Act. Data include the date of disease occurrence, date of testing, date of positive test and diagnoses. The register is held by the Public Health Agency of Sweden.	Register	Nationwide	2020 - today	Daily	No lag	(15)

Supplementary Table S3. Overview of vaccine brands used in the national program in the Nordic countries in season 2024/2025.

Country	Vaccine Brand	Vaccine Type	Target population
Denmark	InfluvacTetra®	QIV, subunit	Risk groups above 6 months
			Individuals 65-69 years
	Vaxigrip Tetra®	QIV, split virion	Risk groups above 6 months
			Individuals 65-69 years, DANFLU-2 clinical trial
	Efluelda Tetra®	QIV, high-dose	DANFLU-2 clinical trial, 65+
	Fluad Tetra®	QIV, adjuvanted	Elderly 70+
	Flucelvax Tetra®	QIV, subunit, cell-based	Individuals with serious allergy to egg, neomycin or gentamycin
Finland	InfluvacTetra®	QIV, subunit	Outside national vaccination program
	Vaxigrip Tetra®	QIV, split virion	All target groups (incl. 2-6-year-olds)
	Fluenz®	Attenuated live virus, nose spray	Children 2-6 years
	Fluad Tetra®	QIV, adjuvanted	Elderly ≥85y
			Severely immunocompromised ≥50y
	Efluelda Tetra®	QIV, high-dose	Outside national vaccination program
Sweden	Vaxigrip Tetra®	QIV, split virion	All target groups (risk groups above 6 months and all above 65)
	InfluvacTetra®	QIV, subunit	All target groups (risk groups above 6 months and all above 65)
	Efluelda Tetra®	QIV, high-dose	Individuals in long term care facilities (nursery homes for elderly) only

Supplementary References

1. Schmidt M, Pedersen L, Sørensen HT. The Danish Civil Registration System as a tool in epidemiology. Eur J Epidemiol. 2014 Aug;29(8):541–9.
2. Grove Krause T, Jakobsen S, Haarh M, Mølbak K. The Danish vaccination register. Euro Surveill. 2012;17(17). Accessed Febr. 2015;16.
3. Schmidt M, Schmidt SAJ, Sandegaard JL, Ehrenstein V, Pedersen L, Sørensen HT. The Danish National Patient Registry: a review of content, data quality, and research potential. Clin Epidemiol [Internet]. 2015 Nov 17 [cited 2023 Feb 15];7:449–90. Available from: https://pubmed.ncbi.nlm.nih.gov/26604824/
4. Schønning K, Dessau RB, Jensen TG, others. Electronic reporting of diagnostic laboratory test results from all healthcare sectors is a cornerstone of national preparedness and control of COVID-19 in Denmark. APMIS. 2021;129(7):438–51.
5. Population Information System | Digital and population data services agency [Internet]. [cited 2024 Aug 29]. Available from: https://dvv.fi/en/population-information-system
6. Baum U, Sundman J, Jääskeläinen S, Nohynek H, Puumalainen T, Jokinen J. Establishing and maintaining the national vaccination register in Finland. Eurosurveillance. 2017 Apr;22(17):30520.
7. Care Register for Health Care - THL [Internet]. Available from: https://thl.fi/en/web/thlfi-en/statistics-and-data/data-and-services/register-descriptions/care-register-for-health-care
8. Register of Primary Health Care visits - THL [Internet]. Available from: https://thl.fi/en/web/thlfi-en/statistics-and-data/data-and-services/register-descriptions/register-of-primary-health-care-visits

9. Finnish National Infectious Diseases Register - THL [Internet]. Finnish Institute for Health and Welfare (THL), Finland. Available from: https://thl.fi/en/web/infectious-diseases-and-vaccinations/surveillance-and-registers/finnish-national-infectious-diseases-register

10. Statistics on reimbursements for medical expenses | Kela's Info Tray [Internet]. [cited 2024 Aug 30]. Available from: https://tietotarjotin.fi/en/statistic/2855002/statistics-on-reimbursements-for-medical-expenses

11. Intensive care quality register (national quality register) - THL [Internet]. [cited 2024 Aug 29]. Available from: https://thl.fi/en/statistics-and-data/data-and-services/register-descriptions/intensive-care-quality-register-national-quality-register-

12. Chrapkowska C, Galanis I, Kark M, Lepp T, Lindstrand A, Roth A, et al. Validation of the new Swedish vaccination register - Accuracy and completeness of register data. Vaccine. 2020 May;38(25):4104–10.

13. Ludvigsson JF, Andersson E, Ekbom A, Feychting M, Kim JL, Reuterwall C, et al. External review and validation of the Swedish national inpatient register. BMC Public Health. 2011 Jun;11:450.

14. Wettermark B, Hammar N, Fored CM, Leimanis A, Olausson PO, Bergman U, et al. The new Swedish Prescribed Drug Register--opportunities for pharmacoepidemiological research and experience from the first six months. Pharmacoepidemiol Drug Saf [Internet]. 2007 [cited 2024 Aug 29];16(7):726–35. Available from: https://pubmed.ncbi.nlm.nih.gov/16897791/

15. Rolfhamre P, Jansson A, Arneborn M, Ekdahl K. SmiNet-2: Description of an internet-based surveillance system for communicable diseases in Sweden. Euro Surveill Bull Eur Sur Les Mal Transm = Eur Commun Dis Bull. 2006;11(5):103–7.
